# Geometric and energetic data from quantum chemical calculations of halobenzenes and xylenes

**DOI:** 10.1016/j.dib.2020.105386

**Published:** 2020-03-05

**Authors:** Sopanant Datta, Taweetham Limpanuparb

**Affiliations:** Science Division, Mahidol University International College, Mahidol University, 999 Phutthamonthon 4, Salaya, Phutthamonthon, Nakhon Pathom 73170, Thailand

**Keywords:** Halobenzene, Xylene, Relative stability, Steric effect

## Abstract

This article presents theoretical data on geometric and energetic features of halobenzenes and xylenes. Data were obtained from *ab initio* geometry optimization and frequency calculations at HF, B3LYP, MP2 and CCSD levels of theory on 6–311++G(d,p) basis set. In total, 1504 structures of halobenzenes, three structures of xylenes and one structure of benzene were generated and processed by custom-made codes in Mathematica. The quantum chemical calculation was completed in Q-Chem software package. Geometric and energetic data of the compounds are presented in this paper as supplementary tables. Raw output files as well as codes and scripts associated with production and extraction of data are also provided.

**Table:** **Specifications Table**

Subject	Chemistry
Specific subject area	Physical and Theoretical Chemistry/Spectroscopy
Type of data	Tables and Q-Chem output files
How data were acquired	Quantum chemical computation on Q-Chem 5.2.1, Developer Version
Data format	Raw and analysed
Parameters for data collection	Hartree-Fock (HF)/6–311++G(d,p), Becke, 3-parameter, Lee–Yang–Parr (B3LYP)/6–311++G(d,p), Second order Møller–Plesset perturbation theory (MP2)/6–311++G(d,p) Coupled Cluster Singles and Doubles (CCSD)/6–311++G(d,p)
Description of data collection	Geometric and energetic data from quantum chemical calculations of halobenzenes, xylenes and benzene were generated by quantum chemical computation and processed by custom-made codes
Data source location	Mahidol University, Salaya, Thailand Latitude and longitude: 13.792790, 100.325707
Data accessibility	With the article

## Value of the data

• All 1505 possible halobenzenes and three xylenes are explicitly shown in this paper with numbering, IUPAC name, PubChem CID and SMILES. These can be used as a reference for both theoretical and experimental work involving this class of compounds.
• Geometric and energetic data can be used for further analysis to understand relative stability of isomers. In particular, the unexpected trend in relative stability of isomers are of particular interest to scientists in a similar manner to cis and gauche effect. The data set includes many examples where steric hindrance alone fails to account for the behaviour observed in halobenzenes and xylenes.
• Raw data as well as associated scripts and codes are provided so that interested researchers can reproduce our data and perform calculation at other levels of theory or for other relevant classes of compounds. Vibrational spectrum and other detailed information can be extracted from output files as needed. There are many potential uses of the spectral information, for example, detection of xylene for food safety application [1] and understanding formation of polychlorinated biphenyls (PCBs) [2]. The data can also be a test set for molecular modelling software packages.

## 1 Data description

**Fig. 1:**
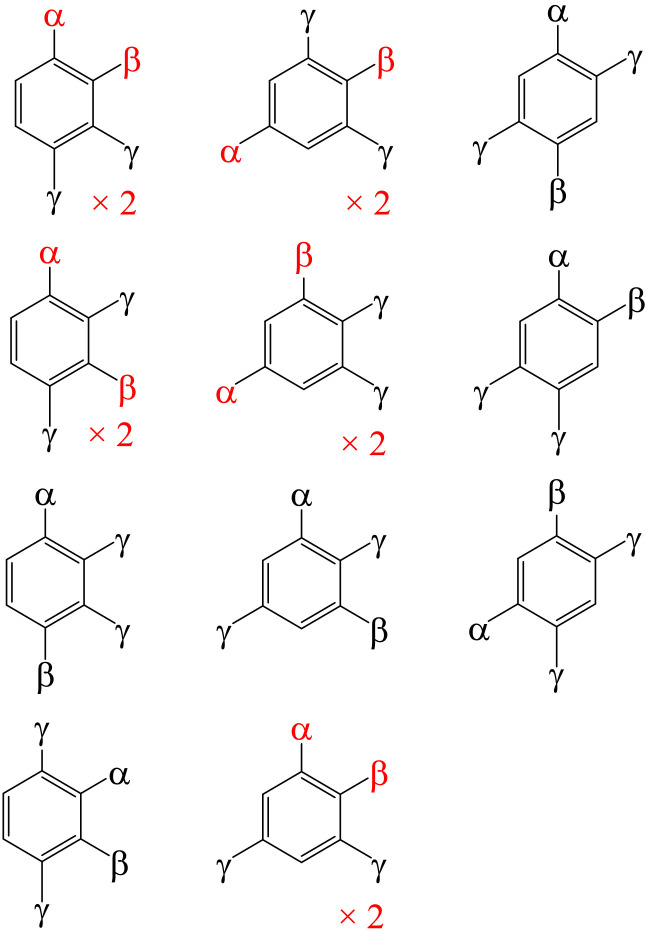
List of 6 + 7 + 3 = 16 structures of halobenzene with empirical formula C_6_αβγ_2_δ_2_ (distribution of elements 1-1-2-2). For simplicity, the two δ are omitted and structures are organised into groups by which from left to right, the first four substituents are in positions 1,2,3,4-, 1,2,3,5- and 1,2,4,5-, respectively. If switching the red letters of a structure leads to a different isomer, then that single depiction represents two different structures as shown with the notation “×2”. Letters α, β, γ, and δ represent different substituents of F, Cl, Br and I. (For Table 1, one of the letters may represent a hydrogen atom.).

**Fig. 2:**
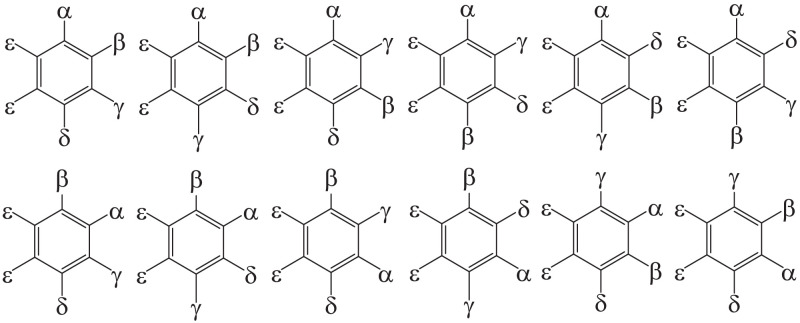
List of halobenzenes with the formula C_6_αβγδε_2_ where permutation of α, β, γ, δ at four adjacent positions (1,2,3,4-) leads to $\frac{4!}{2}=12$ possible structures. The division by two arises due to the symmetry of the structure.

**Fig. 3:**
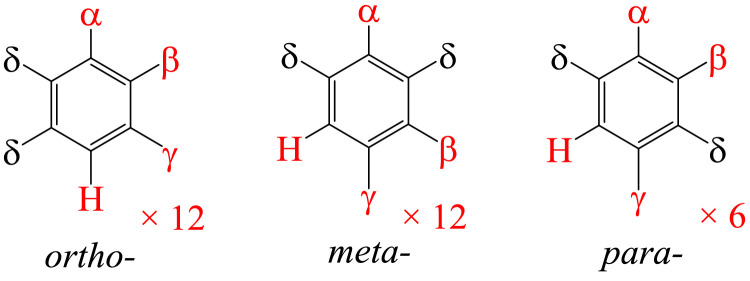
Possible structures of pentahalobenzene C_6_Hαβγδ_2_ with 4 different halogens acting as substituents (distribution of elements: 1-1-1-2). Structures are divided into three groups with 12, 12 and 6 structures due to permutation for δ atoms (any halogen listed but not H) in *ortho*-, *meta*-, and *para*- positions, respectively. A full list of structures of the *ortho* group is shown in Fig. 2. (Reassignment of letters is needed.).

A total of 1505 unique compounds of benzene, including all degrees of substitution with F, Cl, Br and I atoms, and three isomers of xylene were investigated. Classification and counting of the 1505 compounds are exhaustively shown in Tables 1 and 2 with specific examples in Fig. 1, Fig. 2, Fig. 3. The main difference between Tables 1 and 2 is the treatment of hydrogen atom. In Table 1, hydrogen is treated in the same way as halogen and this leads to the binomial coefficients $\left(\begin{array}{c} 5\\ k \end{array}\right)$ for five kinds of elements. In Table 2, hydrogen is treated in a special way and this leads to binomial coefficients $\left(\begin{array}{c} 4\\ k \end{array}\right)$ for four kinds of halogen atoms. Table 3 summarizes the total number of Q-Chem 5.2.1 [3] output files for different classes of compounds, types of calculation (geometry optimization/frequency calculation) and levels of theory (HF, B3LYP, MP2, and CCSD)

**Table 1:** List of all compounds by the number of elements bonded to carbon atoms (In total, there are 1505 benzene and halobenzene compounds with 210 possible empirical formulas.).

Number of elements	Distribution of elements	Number of empirical formulas	Position of elements	Number of isomers per formula	Number of structures
1	C_6_α_6_ (6)	$\left(\begin{array}{c} 5\\ 1 \end{array}\right)=\;$5	n/a	1	5

2	C_6_α_5_β (1–5)	$\left(\begin{array}{c} 5\\ 2 \end{array}\right)\left(\begin{array}{c} 2\\ 1 \end{array}\right)=\;$20	1-	1	20
	C_6_α_2_β_4_ (2–4)	$\left(\begin{array}{c} 5\\ 2 \end{array}\right)\left(\begin{array}{c} 2\\ 1 \end{array}\right)=\;$20	1,2-	1	20
			1,3-	1	20
			1,4-	1	20
	C_6_α_3_β_3_ (3–3)	$\left(\begin{array}{c} 5\\ 2 \end{array}\right)=\;$10	1,2,3-	1	10
			1,2,4-	1	10
			1,3,5-	1	10

3	C_6_αβγ_4_ (1–1–4)	$\left(\begin{array}{c} 5\\ 3 \end{array}\right)\left(\begin{array}{c} 3\\ 1 \end{array}\right)=\;$30	1,2-	1	30
			1,3-	1	30
			1,4-	1	30
	C_6_αβ_2_γ_3_ (1–2–3)	$\left(\begin{array}{c} 5\\ 3 \end{array}\right)\left(\begin{array}{c} 3\\ 1 \end{array}\right)\left(\begin{array}{c} 2\\ 1 \end{array}\right)=$ 60	1,2,3-	2	120
			1,2,4-	3	180
			1,3,5-	1	60
	C_6_α_2_β_2_γ_2_ (2–2–2)	$\left(\begin{array}{c} 5\\ 3 \end{array}\right)=$ 10	1,2,3,4-	4	40
			1,2,3,5-	4	40
			1,2,4,5-	3	30

4	C_6_αβγδ_3_ (1–1–1–3)	$\left(\begin{array}{c} 5\\ 4 \end{array}\right)\left(\begin{array}{c} 4\\ 1 \end{array}\right)=$ 20	1,2,3-	3	60
			1,2,4-	6	120
			1,3,5-	1	20
	C_6_αβγ_2_δ_2_ (1–1–2–2)	$\left(\begin{array}{c} 5\\ 4 \end{array}\right)\left(\begin{array}{c} 4\\ 2 \end{array}\right)=$ 30	1,2,3,4-	**6**^a^	180
			1,2,3,5-	**7**^a^	210
			1,2,4,5-	**3**^a^	90

5	C_6_αβγδε_2_ (1–1–1–1–2)	$\left(\begin{array}{c} 5\\ 5 \end{array}\right)\left(\begin{array}{c} 5\\ 1 \end{array}\right)=$ 5	1,2,3,4-	**12**^b^	60
			1,2,3,5-	12	60
			1,2,4,5-	6	30

a See Fig. 1.

b See Fig. 2.

**Table 2:** List of all compounds by different degrees of substitution to benzene (In total, the number of compounds and empirical formulas is the same as in Table 1).

Group of compounds	Number of halogen substituents	Distribution of substituents	Number of empirical formulas	Position of substituent	Number of isomers per formula	Number of structures
Benzene	0	C_6_H_6_	$\left(\begin{array}{c} 4\\ 0 \end{array}\right)=\;$1	n/a	1	1

Monohalobenzene	1	C_6_H_5_α (1)	$\left(\begin{array}{c} 4\\ 1 \end{array}\right)=\;4$	1	1	4

Dihalobenzene	1	C_6_H_4_α_2_ (2)	$\left(\begin{array}{c} 4\\ 1 \end{array}\right)=\;$4	1,2-	1	4
				1,3-	1	4
				1,4-	1	4
	2	C_6_H_4_αβ (1–1)	$\left(\begin{array}{c} 4\\ 2 \end{array}\right)=\;$6	1,2-	1	6
				1,3-	1	6
				1,4-	1	6

Trihalobenzene	1	C_6_H_3_α_3_ (3)	$\left(\begin{array}{c} 4\\ 1 \end{array}\right)=\;$4	1,2,3-	1	4
				1,2,4-	1	4
				1,3,5-	1	4
	2	C_6_H_3_αβ_2_ (1–2)	$\left(\begin{array}{c} 4\\ 2 \end{array}\right)\left(\begin{array}{c} 2\\ 1 \end{array}\right)\;$= 12	1,2,3-	2	24
				1,2,4-	3	36
				1,3,5-	1	12
	3	C_6_H_3_αβγ (1–1–1)	$\left(\begin{array}{c} 4\\ 1 \end{array}\right)=\;$4	1,2,3-	3	12
				1,2,4-	6	24
				1,3,5-	1	4

Tetrahalobenzene	1	C_6_H_2_α_4_ (4)	$\left(\begin{array}{c} 4\\ 1 \end{array}\right)=\;$4	1,2,3,4-	1	4
				1,2,3,5-	1	4
				1,2,4,5-	1	4
	2	C_6_H_2_αβ_3_ (1–3)	$\left(\begin{array}{c} 4\\ 2 \end{array}\right)\left(\begin{array}{c} 2\\ 1 \end{array}\right)=\;$12	1,2,3,4-	2	24
				1,2,3,5-	3	36
				1,2,4,5-	1	12
		C_6_H_2_α_2_β_2_ (2–2)	$\left(\begin{array}{c} 4\\ 2 \end{array}\right)=\;$6	1,2,3,4-	4	24
				1,2,3,5-	4	24
				1,2,4,5-	3	18
	3	C_6_H_2_αβγ_2_ (1–1–2)	$\left(\begin{array}{c} 4\\ 3 \end{array}\right)\left(\begin{array}{c} 3\\ 1 \end{array}\right)=\;$12	1,2,3,4-	6	72
				1,2,3,5-	7	84
				1,2,4,5-	3	36
	4	C_6_H_2_αβγδ (1–1–1–1)	$\left(\begin{array}{c} 4\\ 4 \end{array}\right)=\;$1	1,2,3,4-	12	12
				1,2,3,5-	12	12
				1,2,4,5-	6	6

Pentahalobenzene	1	C_6_Hα_5_ (5)	$\left(\begin{array}{c} 4\\ 1 \end{array}\right)=\;$4	1,2,3,4,5-	1	4
	2	C_6_Hαβ_4_ (1–4)	$\left(\begin{array}{c} 4\\ 2 \end{array}\right)\left(\begin{array}{c} 2\\ 1 \end{array}\right)=\;$12	1,2,3,4,5-	3	36
		C_6_Hα_2_β_3_ (2–3)	$\left(\begin{array}{c} 4\\ 2 \end{array}\right)\left(\begin{array}{c} 2\\ 1 \end{array}\right)=\;$12	1,2,3,4,5-	6	72
	3	C_6_Hαβγ_3_ (1–1–3)	$\left(\begin{array}{c} 4\\ 3 \end{array}\right)\left(\begin{array}{c} 3\\ 1 \end{array}\right)=\;$12	1,2,3,4,5-	10	120
		C_6_Hαβ_2_γ_2_ (1–2–2)	$\left(\begin{array}{c} 4\\ 3 \end{array}\right)\left(\begin{array}{c} 3\\ 1 \end{array}\right)=\;$12	1,2,3,4,5-	16	192
	4	C_6_Hαβγδ_2_ (1–1–1–2)	$\left(\begin{array}{c} 4\\ 4 \end{array}\right)\left(\begin{array}{c} 4\\ 1 \end{array}\right)=\;$4	1,2,3,4,5-	**30**^a^	120
Hexahalobenzene	1	C_6_α_6_ (6)	$\left(\begin{array}{c} 4\\ 1 \end{array}\right)=\;$4	1,2,3,4,5,6-	1	4
	2	C_6_αβ_5_ (1–5)	$\left(\begin{array}{c} 4\\ 2 \end{array}\right)\left(\begin{array}{c} 2\\ 1 \end{array}\right)=\;$12	1,2,3,4,5,6-	1	12
		C_6_α_2_β_4_ (2–4)	$\left(\begin{array}{c} 4\\ 2 \end{array}\right)\left(\begin{array}{c} 2\\ 1 \end{array}\right)=\;$12	1,2,3,4,5,6-	3	36
		C_6_α_3_β_3_ (3–3)	$\left(\begin{array}{c} 4\\ 2 \end{array}\right)=\;$6	1,2,3,4,5,6-	3	18
	3	C_6_αβγ_4_ (1–1–4)	$\left(\begin{array}{c} 4\\ 3 \end{array}\right)\left(\begin{array}{c} 3\\ 1 \end{array}\right)=\;$12	1,2,3,4,5,6-	3	36
		C_6_αβ_2_γ_3_ (1–2–3)	$\left(\begin{array}{c} 4\\ 3 \end{array}\right)\left(\begin{array}{c} 3\\ 1 \end{array}\right)\left(\begin{array}{c} 2\\ 1 \end{array}\right)=\;$24	1,2,3,4,5,6-	6	144
		C_6_α_2_β_2_γ_2_ (2–2–2)	$\left(\begin{array}{c} 4\\ 3 \end{array}\right)=\;$4	1,2,3,4,5,6-	11	44
	4	C_6_αβγδ_3_ (1–1–1–3)	$\left(\begin{array}{c} 4\\ 4 \end{array}\right)\left(\begin{array}{c} 4\\ 1 \end{array}\right)=\;$4	1,2,3,4,5,6-	10	40
		C_6_αβγ_2_δ_2_ (1–1–2–2)	$\left(\begin{array}{c} 4\\ 4 \end{array}\right)\left(\begin{array}{c} 4\\ 2 \end{array}\right)=\;$6	1,2,3,4,5,6-	**16**^b^	96

a See Fig. 3.

b See Fig. 1.

**Table 3:** Summary of investigated compounds, levels of theory (HF, B3LYP, MP2, and CCSD) on 6–311++G(d,p) basis set and types of calculation (opt for geometry optimization and freq for frequency calculation).

Group of compounds	Number of tuples	Number of structures	HF		B3LYP		MP2		CCSD	
			opt	freq	opt	freq	opt	freq	opt	freq
Benzene	1	1	all	all	all	all	all	–	all	–
Monohalobenzene	24	4	all	all	all	all	all	–	all	–
Dihalobenzene	240	30	all	all	all	all	all	–	all	–
Trihalobenzene	1280	124	all	all	all	all	all	–	–	–
Tetrahalobenzene	3840	372	all	all	all	all	all	–	–	–
Pentahalobenzene	6144	544	all	all	all	all	all	–	–	–
Hexahalobenzene	4096	430	all	all	all	all	all	–	–	–
Xylene	15	3	all	all	all	all	all	–	all	–
Total	15,640	1508	1508	1508	1508	1508	1508	–	38	–

In supplementary information, summary table files (.csv) are provided per level of theory.

• Geometric data of 12 bond lengths, 12 bond angles and 12 torsional angles in a single csv file
• Energetic data, in separate files, include electronic energy (*E*_elec_) in Hartree, thermal correction to enthalpy (*H*_corr_) in kcal mol^−1^, zero-point vibrational energy (*E*_ZPE_) in kcal mol^−1^ and entropy (*S*) in cal mol^−1^ K^−1^.

The following associated files are also provided.

• Raw Q-Chem output files (.out) for all compounds.
• Geometry in Z-matrix and Cartesian coordinate format (.xyz) for all compounds.
• Wolfram Mathematica notebook (benzene.nb) and associated script (script.txt).

## 2 Experimental design, materials, and methods

Due to prohibitive computational cost, frequency calculations at MP2 and CCSD levels of theory were excluded and only benzene to dihalobenzenes and xylenes were selected for CCSD optimization jobs. The output files were processed by custom-made scripts and Wolfram Mathematica 12.0 [4] codes to extract geometric and energetic data of all halobenzene compounds in a similar manner to our previous work [5]. Data from the three xylene compounds are provided for reference purpose and were read from IQmol 2.13 manually [6].

## Appendix Supplementary materials

Image, application 1

Image, application 2

Image, application 3
